# (2*E*)-1-(4,4′′-Difluoro-5′-meth­oxy-1,1′:3′,1′′-terphenyl-4′-yl)-3-(4-fluoro­phen­yl)prop-2-en-1-one

**DOI:** 10.1107/S1600536811042279

**Published:** 2011-10-22

**Authors:** Richard Betz, Thomas Gerber, Eric Hosten, S. Samshuddin, Badiadka Narayana, Balladka K. Sarojini

**Affiliations:** a Nelson Mandela Metropolitan University, Summerstrand Campus, Department of Chemistry, University Way, Summerstrand, PO Box 77000, Port Elizabeth 6031, South Africa; b Mangalore University, Department of Studies in Chemistry, Mangalagangotri 574 199, India; c P.A. College of Engineering, Department of Chemistry, Nadupadavu, Mangalore 574 199, India

## Abstract

In the title compound, C_28_H_19_F_3_O_2_, the C=C double bond has an *E* configuration. In the crystal, C—H⋯F contacts link the mol­ecules into chains along [111]. The shortest centroid–centroid distance between two π systems is 3.8087 (8) Å and is apparent between the *para*-fluoro­phenyl group attached to the Michael system and its symmetry-generated equivalent.

## Related literature

For the pharmacological importance of terphenyls, see: Liu (2006 ▶) and of chalcones, see: Dhar (1981 ▶); Dimmock *et al.* (1999 ▶); Satyanarayana *et al.* (2004 ▶). For work on the synthesis and strcutures of different chalcone derivatives, see: Samshuddin *et al.* (2011*a* ▶,*b* ▶); Fun *et al.* (2010*a* ▶,*b* ▶); Jasinski *et al.* (2010*a* ▶,*b* ▶); Baktir *et al.* (2011*a* ▶,*b* ▶). For graph-set analysis of hydrogen bonds, see: Etter *et al.* (1990 ▶); Bernstein *et al.* (1995 ▶).
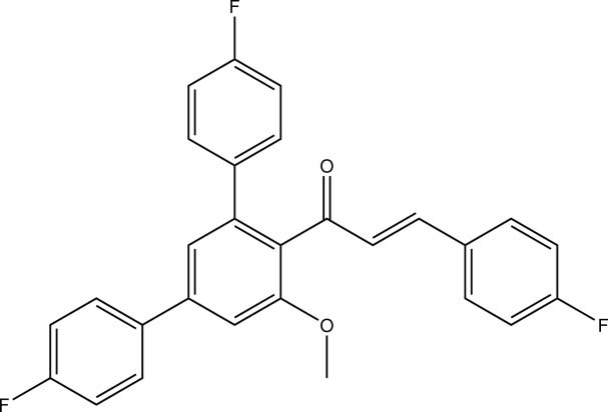


## Experimental

### 

#### Crystal data

C_28_H_19_F_3_O_2_
*M* *_r_* = 444.43
Monoclinic, 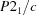

*a* = 22.5742 (5) Å
*b* = 6.8101 (1) Å
*c* = 13.8475 (3) Å
β = 98.405 (1)°
*V* = 2105.95 (7) Å^3^
*Z* = 4
Mo *K*α radiation
μ = 0.11 mm^−1^
*T* = 200 K
0.56 × 0.26 × 0.18 mm

#### Data collection

Bruker APEXII CCD diffractometer
35196 measured reflections
5218 independent reflections
4087 reflections with *I* > 2σ(*I*)
*R*_int_ = 0.032

#### Refinement

*R*[*F* ^2^ > 2σ(*F* ^2^)] = 0.038
*wR*(*F* ^2^) = 0.102
*S* = 1.04
5218 reflections
299 parameters
H-atom parameters constrained
Δρ_max_ = 0.30 e Å^−3^
Δρ_min_ = −0.19 e Å^−3^

### 

Data collection: *APEX2* (Bruker, 2010 ▶); cell refinement: *SAINT* (Bruker, 2010 ▶); data reduction: *SAINT*; program(s) used to solve structure: *SHELXS97* (Sheldrick, 2008 ▶); program(s) used to refine structure: *SHELXL97* (Sheldrick, 2008 ▶); molecular graphics: *ORTEP-3* (Farrugia, 1997 ▶) and *Mercury* (Macrae *et al.*, 2008 ▶); software used to prepare material for publication: *SHELXL97* and *PLATON* (Spek, 2009 ▶).

## Supplementary Material

Crystal structure: contains datablock(s) I, global. DOI: 10.1107/S1600536811042279/zl2413sup1.cif

Structure factors: contains datablock(s) I. DOI: 10.1107/S1600536811042279/zl2413Isup2.hkl

Supplementary material file. DOI: 10.1107/S1600536811042279/zl2413Isup3.cdx

Supplementary material file. DOI: 10.1107/S1600536811042279/zl2413Isup4.cml

Additional supplementary materials:  crystallographic information; 3D view; checkCIF report

## Figures and Tables

**Table 1:** Hydrogen-bond geometry (Å, °)

*D*—H⋯*A*	*D*—H	H⋯*A*	*D*⋯*A*	*D*—H⋯*A*
C25—H25⋯F1^i^	0.95	2.55	3.2276 (15)	129
C43—H43⋯F2^ii^	0.95	2.53	3.4449 (16)	161

Symmetry codes: (i) 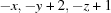; (ii) 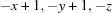.

**Fig. 1.**
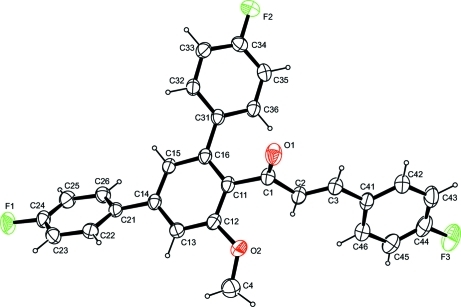
The molecular structure of the title compound, with atom labels and anisotropic displacement ellipsoids (drawn at 50% probability level).

**Fig. 2.**
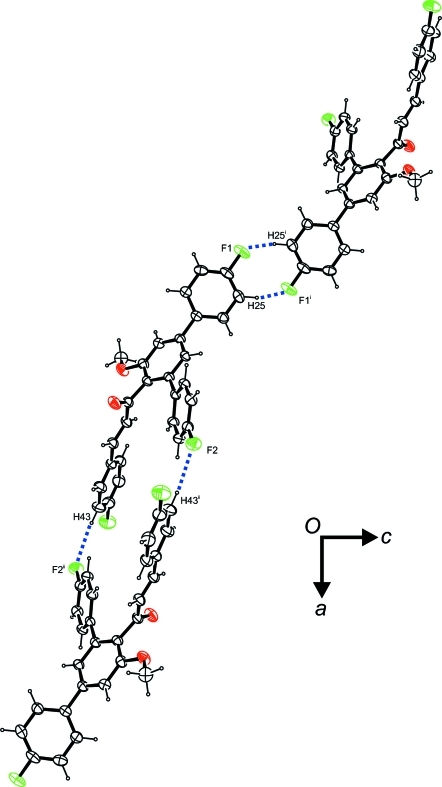
Intermolecular contacts, viewed along [0 1 0]. Symmetry operators: i -x, -y + 2, -z + 1; ii -x + 1, -y + 1, -z.

**Fig. 3.**
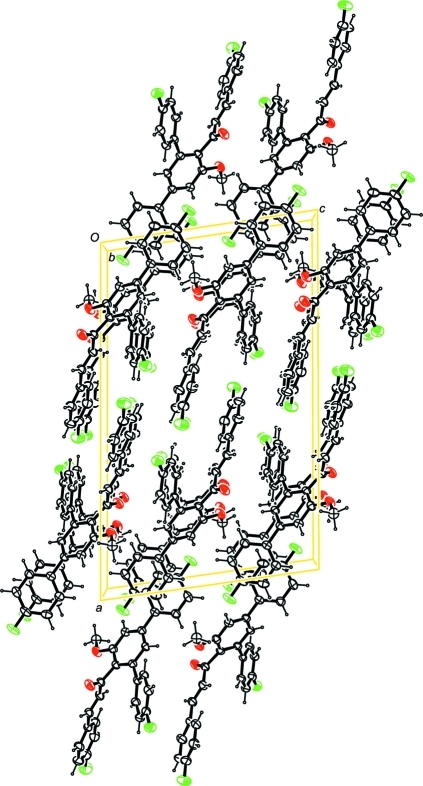
Molecular packing of the title compound, viewed along [0 1 0] (anisotropic displacement ellipsoids drawn at 50% probability level).

## supplementary crystallographic information

### Comment

Chalcones constitute an important family of substances belonging to flavonoids, a large group of natural and synthetic products with interesting physicochemical properties, biological activity and structural characteristics. They have been reported to possess many interesting pharmacological activities (Dhar, 1981) including anti-inflammatory, antimicrobial, antifungal, antioxidant, cytotoxic, antitumor and anticancer activities (Dimmock *et al.*, 1999; Satyanarayana *et al.*, 2004). In recent years, it has been reported that some terphenyls exhibit considerable biological activities (*e.g.* being potent anticoagulants, immunosuppressants, antithrombotics, neuroprotectives, specific 5-lipoxygenase inhibitors) and showing cytotoxic activities (Liu, 2006). In view of the pharmacological importance of terphenyls and chalcones, and in continuation of our work on synthesis of various derivatives of 4,4'-difluoro chalcone (Samshuddin *et al.*, 2011*a*/b, Fun *et al.*, 2010*a*/b, Jasinski *et al.*, 2010*a*/b, Baktir *et al.*, 2011*a*/b), the molecular and crystal structure of the title compound is reported.

The C=C double of the Michael system is (*E*)-configured. The least-squares planes defined by the carbon atoms of the *para*-fluoro phenyl rings of the terphenyl moiety and its central phenyl ring enclose angles of 40.37 (6)° and 44.04 (6)°, respectively (Fig. 1).

In the crystal, two different C–H···F contacts whose range falls by more than 0.1 Å below the sum of van-der-Waals radii of the atoms participating are observed. The first contact is apparent between one of the fluorine atoms on the terphenyl skeleton and a hydrogen atom on the terminal aromatic substituent on the Michael system's substituent. The second one is supported by the second fluorine atom on the terphenyl skeleton and a hydrogen atom on its symmetry-generated equivalent. Both contacts connect the molecules in such a way that cyclic patterns with local inversion symmetry are generated. In terms of graph-set analysis (Etter *et al.*, 1990; Bernstein *et al.*, 1995), the descriptor for the C–H···F contacts is *R*^2^_2_(8)*R*^2^_2_(28) on the unitary level. In total, the molecules are connected to infinite chains along [1 1 1]. Metrical parameters as well as information about the symmetry of these contacts is summarized in Table 1. The shortest intercentroid distance between two π systems was found at 3.8087 (8) Å and is apparent between the *para*-fluoro phenyl moiety attached to the Michael system and its symmetry-generated equivalent. (Fig. 2).

The packing of the title compound in the crystal is shown in Figure 3.

### Experimental

To a mixture of 1-(4,4''-difluoro-5'-methoxy-1,1':3',1''-terphenyl-4'-yl) ethanone (0.338 g, 0.001 mol) and *p*-fluorobenzaldehyde (0.124 g, 0.001 mol) in 30 ml e thanol, 1 ml of 10% sodium hydroxide solution was added and stirred at 278–283 K for 3 h. The precipitate formed was collected by filtration and purified by recrystallization from ethanol (yield: 83%). Single crystals suitable for the X-ray diffraction study were grown from a 1:1 (*v*:*v*) mixture of DMF and ethanol by slow evaporation at room temperature.

### Refinement

Carbon-bound H atoms were placed in calculated positions (C—H 0.95 Å for aromatic and vinylic carbon atoms, C—H 0.99 Å for methylene groups) and were included in the refinement in the riding model approximation, with *U*(H) set to 1.2*U*_eq_(C). The H atoms of the methyl groups were allowed to rotate with a fixed angle around the C—C bond to best fit the experimental electron density (HFIX 137 in the *SHELX* program suite (Sheldrick, 2008)), with *U*(H) set to 1.5*U*_eq_(C).

### Crystal data

**Table:**

C_28_H_19_F_3_O_2_	*F*(000) = 920
*M**_r_* = 444.43	*D*_x_ = 1.402 Mg m^−^^3^
Monoclinic, *P*2_1_/*c*	Melting point: 549 K
Hall symbol: -P 2ybc	Mo *K*α radiation, λ = 0.71073 Å
*a* = 22.5742 (5) Å	Cell parameters from 9830 reflections
*b* = 6.8101 (1) Å	θ = 3.0–28.3°
*c* = 13.8475 (3) Å	µ = 0.11 mm^−^^1^
β = 98.405 (1)°	*T* = 200 K
*V* = 2105.95 (7) Å^3^	Block, yellow
*Z* = 4	0.56 × 0.26 × 0.18 mm

### Data collection

**Table:**

Bruker APEXII CCD diffractometer	4087 reflections with *I* > 2σ(*I*)
Radiation source: fine-focus sealed tube	*R*_int_ = 0.032
graphite	θ_max_ = 28.3°, θ_min_ = 1.8°
φ and ω scans	*h* = −30→30
35196 measured reflections	*k* = −9→8
5218 independent reflections	*l* = −18→18

### Refinement

**Table:**

Refinement on *F*^2^	Primary atom site location: structure-invariant direct methods
Least-squares matrix: full	Secondary atom site location: difference Fourier map
*R*[*F*^2^ > 2σ(*F*^2^)] = 0.038	Hydrogen site location: inferred from neighbouring sites
*wR*(*F*^2^) = 0.102	H-atom parameters constrained
*S* = 1.04	*w* = 1/[σ^2^(*F*_o_^2^) + (0.0462*P*)^2^ + 0.5814*P*] where *P* = (*F*_o_^2^ + 2*F*_c_^2^)/3
5218 reflections	(Δ/σ)_max_ < 0.001
299 parameters	Δρ_max_ = 0.30 e Å^−^^3^
0 restraints	Δρ_min_ = −0.19 e Å^−^^3^

### Fractional atomic coordinates and isotropic or equivalent isotropic displacement parameters (Å^2^)

**Table:**

	*x*	*y*	*z*	*U*_iso_*/*U*_eq_	
F1	−0.04953 (4)	0.88945 (14)	0.40789 (7)	0.0567 (3)	
F2	0.37032 (4)	0.00783 (12)	0.22814 (6)	0.0486 (2)	
F3	0.53866 (4)	1.35725 (17)	−0.11810 (8)	0.0755 (3)	
O1	0.25624 (4)	0.57595 (14)	−0.06967 (7)	0.0445 (2)	
O2	0.18161 (4)	1.02710 (14)	−0.05061 (7)	0.0422 (2)	
C1	0.25957 (5)	0.72789 (19)	−0.02279 (8)	0.0317 (3)	
C2	0.30451 (6)	0.88085 (19)	−0.03214 (9)	0.0343 (3)	
H2	0.3015	1.0037	−0.0007	0.041*	
C3	0.34907 (5)	0.85151 (19)	−0.08335 (9)	0.0342 (3)	
H3	0.3493	0.7298	−0.1168	0.041*	
C4	0.15353 (7)	1.2144 (2)	−0.06059 (10)	0.0455 (3)	
H4A	0.1662	1.2924	−0.0017	0.068*	
H4B	0.1100	1.1977	−0.0695	0.068*	
H4C	0.1651	1.2821	−0.1175	0.068*	
C11	0.21736 (5)	0.76519 (17)	0.05006 (8)	0.0291 (2)	
C12	0.17663 (5)	0.92003 (18)	0.03161 (9)	0.0313 (3)	
C13	0.13322 (5)	0.95422 (18)	0.09070 (9)	0.0310 (3)	
H13	0.1050	1.0572	0.0756	0.037*	
C14	0.13129 (5)	0.83612 (17)	0.17253 (8)	0.0285 (2)	
C15	0.17348 (5)	0.68782 (17)	0.19389 (8)	0.0287 (2)	
H15	0.1733	0.6120	0.2514	0.034*	
C16	0.21618 (5)	0.64726 (17)	0.13284 (8)	0.0279 (2)	
C21	0.08371 (5)	0.86298 (16)	0.23502 (9)	0.0289 (2)	
C22	0.02421 (5)	0.89054 (18)	0.19403 (9)	0.0331 (3)	
H22	0.0143	0.9020	0.1252	0.040*	
C23	−0.02064 (6)	0.90140 (18)	0.25208 (10)	0.0375 (3)	
H23	−0.0612	0.9206	0.2240	0.045*	
C24	−0.00510 (6)	0.88379 (18)	0.35085 (10)	0.0384 (3)	
C25	0.05286 (6)	0.8598 (2)	0.39518 (10)	0.0398 (3)	
H25	0.0622	0.8508	0.4642	0.048*	
C26	0.09725 (6)	0.84925 (18)	0.33614 (9)	0.0345 (3)	
H26	0.1377	0.8323	0.3652	0.041*	
C31	0.25833 (5)	0.48112 (17)	0.15868 (8)	0.0274 (2)	
C32	0.23728 (5)	0.30657 (18)	0.19374 (9)	0.0312 (3)	
H32	0.1962	0.2967	0.2010	0.037*	
C33	0.27442 (6)	0.14721 (18)	0.21833 (9)	0.0343 (3)	
H33	0.2596	0.0294	0.2426	0.041*	
C34	0.33354 (6)	0.16525 (18)	0.20640 (9)	0.0333 (3)	
C35	0.35695 (5)	0.3347 (2)	0.17383 (9)	0.0352 (3)	
H35	0.3982	0.3430	0.1673	0.042*	
C36	0.31926 (5)	0.49307 (18)	0.15071 (9)	0.0322 (3)	
H36	0.3350	0.6119	0.1290	0.039*	
C41	0.39775 (5)	0.9881 (2)	−0.09336 (9)	0.0340 (3)	
C42	0.44727 (6)	0.9221 (2)	−0.13332 (10)	0.0425 (3)	
H42	0.4484	0.7899	−0.1550	0.051*	
C43	0.49492 (6)	1.0455 (3)	−0.14204 (11)	0.0504 (4)	
H43	0.5288	0.9998	−0.1690	0.060*	
C44	0.49179 (6)	1.2347 (3)	−0.11076 (11)	0.0498 (4)	
C45	0.44382 (7)	1.3083 (2)	−0.07209 (10)	0.0473 (3)	
H45	0.4431	1.4414	−0.0516	0.057*	
C46	0.39656 (6)	1.1840 (2)	−0.06367 (9)	0.0394 (3)	
H46	0.3628	1.2322	−0.0374	0.047*	

### Atomic displacement parameters (Å^2^)

**Table:**

	*U*^11^	*U*^22^	*U*^33^	*U*^12^	*U*^13^	*U*^23^
F1	0.0539 (5)	0.0573 (6)	0.0682 (6)	−0.0014 (4)	0.0407 (4)	−0.0098 (4)
F2	0.0448 (5)	0.0415 (5)	0.0603 (5)	0.0126 (4)	0.0102 (4)	0.0063 (4)
F3	0.0552 (6)	0.0899 (8)	0.0845 (7)	−0.0380 (5)	0.0209 (5)	0.0072 (6)
O1	0.0498 (6)	0.0425 (5)	0.0458 (5)	−0.0121 (4)	0.0220 (4)	−0.0141 (4)
O2	0.0514 (6)	0.0377 (5)	0.0416 (5)	0.0052 (4)	0.0203 (4)	0.0118 (4)
C1	0.0316 (6)	0.0353 (6)	0.0295 (6)	−0.0033 (5)	0.0092 (5)	−0.0003 (5)
C2	0.0372 (6)	0.0345 (6)	0.0334 (6)	−0.0059 (5)	0.0125 (5)	−0.0022 (5)
C3	0.0348 (6)	0.0366 (7)	0.0327 (6)	−0.0028 (5)	0.0096 (5)	0.0012 (5)
C4	0.0596 (9)	0.0370 (7)	0.0394 (7)	0.0012 (6)	0.0055 (6)	0.0090 (6)
C11	0.0291 (6)	0.0289 (6)	0.0308 (6)	−0.0061 (4)	0.0092 (4)	−0.0028 (5)
C12	0.0347 (6)	0.0288 (6)	0.0317 (6)	−0.0053 (5)	0.0093 (5)	0.0011 (5)
C13	0.0309 (6)	0.0272 (6)	0.0359 (6)	−0.0006 (5)	0.0080 (5)	−0.0006 (5)
C14	0.0283 (6)	0.0271 (6)	0.0313 (6)	−0.0057 (4)	0.0085 (4)	−0.0040 (4)
C15	0.0298 (6)	0.0285 (6)	0.0295 (5)	−0.0044 (5)	0.0094 (4)	−0.0002 (5)
C16	0.0275 (5)	0.0268 (6)	0.0305 (6)	−0.0056 (4)	0.0075 (4)	−0.0033 (4)
C21	0.0305 (6)	0.0228 (5)	0.0351 (6)	−0.0024 (4)	0.0113 (5)	−0.0027 (5)
C22	0.0349 (6)	0.0289 (6)	0.0367 (6)	0.0008 (5)	0.0090 (5)	−0.0008 (5)
C23	0.0310 (6)	0.0302 (6)	0.0534 (8)	0.0008 (5)	0.0129 (6)	−0.0041 (6)
C24	0.0425 (7)	0.0290 (6)	0.0498 (8)	−0.0018 (5)	0.0273 (6)	−0.0070 (5)
C25	0.0493 (8)	0.0382 (7)	0.0348 (6)	0.0004 (6)	0.0163 (6)	−0.0049 (5)
C26	0.0351 (6)	0.0342 (6)	0.0355 (6)	−0.0002 (5)	0.0090 (5)	−0.0046 (5)
C31	0.0286 (6)	0.0287 (6)	0.0260 (5)	−0.0036 (4)	0.0082 (4)	−0.0045 (4)
C32	0.0295 (6)	0.0325 (6)	0.0335 (6)	−0.0035 (5)	0.0109 (5)	−0.0020 (5)
C33	0.0387 (7)	0.0291 (6)	0.0365 (6)	−0.0036 (5)	0.0102 (5)	0.0008 (5)
C34	0.0357 (6)	0.0325 (6)	0.0318 (6)	0.0048 (5)	0.0055 (5)	−0.0028 (5)
C35	0.0270 (6)	0.0419 (7)	0.0376 (6)	−0.0010 (5)	0.0083 (5)	−0.0023 (5)
C36	0.0300 (6)	0.0329 (6)	0.0349 (6)	−0.0057 (5)	0.0090 (5)	−0.0005 (5)
C41	0.0303 (6)	0.0427 (7)	0.0301 (6)	−0.0032 (5)	0.0081 (5)	0.0049 (5)
C42	0.0388 (7)	0.0474 (8)	0.0442 (7)	0.0004 (6)	0.0156 (6)	0.0041 (6)
C43	0.0335 (7)	0.0685 (10)	0.0521 (8)	−0.0030 (7)	0.0164 (6)	0.0089 (8)
C44	0.0392 (7)	0.0645 (10)	0.0459 (8)	−0.0186 (7)	0.0070 (6)	0.0121 (7)
C45	0.0503 (8)	0.0474 (8)	0.0445 (8)	−0.0137 (7)	0.0075 (6)	0.0031 (6)
C46	0.0383 (7)	0.0447 (7)	0.0367 (7)	−0.0048 (6)	0.0106 (5)	0.0014 (6)

### Geometric parameters (Å, °)

**Table:**

F1—C24	1.3648 (14)	C22—H22	0.9500
F2—C34	1.3624 (14)	C23—C24	1.367 (2)
F3—C44	1.3628 (16)	C23—H23	0.9500
O1—C1	1.2180 (15)	C24—C25	1.371 (2)
O2—C12	1.3704 (14)	C25—C26	1.3847 (17)
O2—C4	1.4218 (17)	C25—H25	0.9500
C1—C2	1.4729 (17)	C26—H26	0.9500
C1—C11	1.5070 (16)	C31—C32	1.3936 (16)
C2—C3	1.3278 (17)	C31—C36	1.3981 (16)
C2—H2	0.9500	C32—C33	1.3832 (17)
C3—C41	1.4617 (17)	C32—H32	0.9500
C3—H3	0.9500	C33—C34	1.3741 (18)
C4—H4A	0.9800	C33—H33	0.9500
C4—H4B	0.9800	C34—C35	1.3728 (18)
C4—H4C	0.9800	C35—C36	1.3818 (18)
C11—C12	1.3979 (17)	C35—H35	0.9500
C11—C16	1.4030 (16)	C36—H36	0.9500
C12—C13	1.3849 (17)	C41—C42	1.3925 (18)
C13—C14	1.3954 (17)	C41—C46	1.3974 (19)
C13—H13	0.9500	C42—C43	1.384 (2)
C14—C15	1.3900 (16)	C42—H42	0.9500
C14—C21	1.4859 (15)	C43—C44	1.365 (2)
C15—C16	1.3995 (16)	C43—H43	0.9500
C15—H15	0.9500	C44—C45	1.371 (2)
C16—C31	1.4880 (16)	C45—C46	1.3802 (19)
C21—C26	1.3920 (17)	C45—H45	0.9500
C21—C22	1.3923 (17)	C46—H46	0.9500
C22—C23	1.3836 (18)		
			
C12—O2—C4	117.93 (10)	F1—C24—C25	118.55 (12)
O1—C1—C2	122.93 (11)	C23—C24—C25	123.16 (12)
O1—C1—C11	120.28 (11)	C24—C25—C26	117.86 (12)
C2—C1—C11	116.78 (10)	C24—C25—H25	121.1
C3—C2—C1	121.72 (12)	C26—C25—H25	121.1
C3—C2—H2	119.1	C25—C26—C21	121.26 (12)
C1—C2—H2	119.1	C25—C26—H26	119.4
C2—C3—C41	126.41 (12)	C21—C26—H26	119.4
C2—C3—H3	116.8	C32—C31—C36	117.82 (11)
C41—C3—H3	116.8	C32—C31—C16	119.58 (10)
O2—C4—H4A	109.5	C36—C31—C16	122.59 (10)
O2—C4—H4B	109.5	C33—C32—C31	121.98 (11)
H4A—C4—H4B	109.5	C33—C32—H32	119.0
O2—C4—H4C	109.5	C31—C32—H32	119.0
H4A—C4—H4C	109.5	C34—C33—C32	117.69 (11)
H4B—C4—H4C	109.5	C34—C33—H33	121.2
C12—C11—C16	119.38 (10)	C32—C33—H33	121.2
C12—C11—C1	117.91 (10)	F2—C34—C35	118.87 (11)
C16—C11—C1	122.67 (11)	F2—C34—C33	118.31 (11)
O2—C12—C13	123.54 (11)	C35—C34—C33	122.81 (12)
O2—C12—C11	114.93 (10)	C34—C35—C36	118.60 (11)
C13—C12—C11	121.48 (11)	C34—C35—H35	120.7
C12—C13—C14	119.45 (11)	C36—C35—H35	120.7
C12—C13—H13	120.3	C35—C36—C31	121.05 (11)
C14—C13—H13	120.3	C35—C36—H36	119.5
C15—C14—C13	119.33 (10)	C31—C36—H36	119.5
C15—C14—C21	119.78 (10)	C42—C41—C46	118.44 (12)
C13—C14—C21	120.87 (11)	C42—C41—C3	119.38 (12)
C14—C15—C16	121.73 (11)	C46—C41—C3	122.19 (12)
C14—C15—H15	119.1	C43—C42—C41	121.19 (14)
C16—C15—H15	119.1	C43—C42—H42	119.4
C15—C16—C11	118.53 (11)	C41—C42—H42	119.4
C15—C16—C31	118.53 (10)	C44—C43—C42	117.95 (14)
C11—C16—C31	122.95 (10)	C44—C43—H43	121.0
C26—C21—C22	118.43 (11)	C42—C43—H43	121.0
C26—C21—C14	120.42 (11)	F3—C44—C43	118.54 (14)
C22—C21—C14	121.03 (11)	F3—C44—C45	118.12 (15)
C23—C22—C21	120.96 (12)	C43—C44—C45	123.35 (13)
C23—C22—H22	119.5	C44—C45—C46	118.22 (15)
C21—C22—H22	119.5	C44—C45—H45	120.9
C24—C23—C22	118.31 (12)	C46—C45—H45	120.9
C24—C23—H23	120.8	C45—C46—C41	120.84 (13)
C22—C23—H23	120.8	C45—C46—H46	119.6
F1—C24—C23	118.29 (12)	C41—C46—H46	119.6
			
O1—C1—C2—C3	−8.7 (2)	C22—C23—C24—C25	1.34 (19)
C11—C1—C2—C3	170.25 (12)	F1—C24—C25—C26	178.35 (11)
C1—C2—C3—C41	−176.55 (12)	C23—C24—C25—C26	−1.3 (2)
O1—C1—C11—C12	−114.77 (14)	C24—C25—C26—C21	0.17 (19)
C2—C1—C11—C12	66.28 (15)	C22—C21—C26—C25	0.88 (18)
O1—C1—C11—C16	62.99 (16)	C14—C21—C26—C25	−175.32 (11)
C2—C1—C11—C16	−115.96 (13)	C15—C16—C31—C32	40.84 (15)
C4—O2—C12—C13	19.08 (18)	C11—C16—C31—C32	−139.22 (12)
C4—O2—C12—C11	−163.68 (11)	C15—C16—C31—C36	−138.15 (12)
C16—C11—C12—O2	179.64 (10)	C11—C16—C31—C36	41.80 (17)
C1—C11—C12—O2	−2.53 (15)	C36—C31—C32—C33	−1.33 (17)
C16—C11—C12—C13	−3.06 (17)	C16—C31—C32—C33	179.63 (11)
C1—C11—C12—C13	174.78 (11)	C31—C32—C33—C34	−0.47 (18)
O2—C12—C13—C14	179.37 (11)	C32—C33—C34—F2	−178.47 (11)
C11—C12—C13—C14	2.30 (18)	C32—C33—C34—C35	1.67 (19)
C12—C13—C14—C15	0.79 (17)	F2—C34—C35—C36	179.18 (11)
C12—C13—C14—C21	−177.28 (11)	C33—C34—C35—C36	−0.96 (19)
C13—C14—C15—C16	−3.16 (17)	C34—C35—C36—C31	−0.97 (18)
C21—C14—C15—C16	174.93 (10)	C32—C31—C36—C35	2.07 (17)
C14—C15—C16—C11	2.39 (17)	C16—C31—C36—C35	−178.93 (11)
C14—C15—C16—C31	−177.66 (10)	C2—C3—C41—C42	167.43 (13)
C12—C11—C16—C15	0.71 (16)	C2—C3—C41—C46	−12.3 (2)
C1—C11—C16—C15	−177.02 (10)	C46—C41—C42—C43	1.2 (2)
C12—C11—C16—C31	−179.24 (10)	C3—C41—C42—C43	−178.53 (13)
C1—C11—C16—C31	3.03 (17)	C41—C42—C43—C44	−0.4 (2)
C15—C14—C21—C26	41.88 (16)	C42—C43—C44—F3	179.40 (13)
C13—C14—C21—C26	−140.05 (12)	C42—C43—C44—C45	−0.5 (2)
C15—C14—C21—C22	−134.22 (12)	F3—C44—C45—C46	−179.36 (13)
C13—C14—C21—C22	43.84 (16)	C43—C44—C45—C46	0.6 (2)
C26—C21—C22—C23	−0.87 (18)	C44—C45—C46—C41	0.3 (2)
C14—C21—C22—C23	175.31 (11)	C42—C41—C46—C45	−1.16 (19)
C21—C22—C23—C24	−0.20 (18)	C3—C41—C46—C45	178.57 (12)
C22—C23—C24—F1	−178.34 (11)		

### Hydrogen-bond geometry (Å, °)

**Table:**

*D*—H···*A*	*D*—H	H···*A*	*D*···*A*	*D*—H···*A*
C25—H25···F1^i^	0.95	2.55	3.2276 (15)	129.
C43—H43···F2^ii^	0.95	2.53	3.4449 (16)	161.

Symmetry codes: (i) −*x*, −*y*+2, −*z*+1; (ii) −*x*+1, −*y*+1, −*z*.
